# Commentary on: The Sequence and Reconstructive Modality of Breast Cancer Treatments Affects Wait Times to Adjunctive Therapies in Patients Undergoing Mastectomy with Immediate Breast Reconstruction

**DOI:** 10.1177/22925503251334564

**Published:** 2025-08-11

**Authors:** Ron Barry Somogyi

**Affiliations:** 1 7938 University of Toronto, Toronto, ON, Canada; 2 North York General Hospital, Toronto, ON, Canada

This is a well-written manuscript that presents a well-executed study on an important topic.^1^ As plastic surgeons, we often focus on modifications and refinements in technique.^2^ This is certainly true in the field of immediate breast reconstruction where significant advancements have reduced complication rates to low levels, as demonstrated by this group's results and many others. However, this study highlights an outcome that is more process-dependent than technique-dependent. Coordinating care for breast reconstruction patients who require multiple treatment modalities across specialties is like conducting a symphony with multiple orchestras and this study does an excellent job of quantifying that complexity.

The authors identify several process modifications that may improve the timing between stages of treatment. I would like to highlight and expand on these. First, education is paramount. Every member of the multidisciplinary team and their staff must understand the timing requirements for each stage so they can adjust scheduling in already over-capacity clinics and surgical centers. Second, communication is equally critical. The ability of specialists to communicate efficiently and in real-time—not relying on transcribed and delayed notes—is essential in keeping patients on track. On a larger scale, as the authors emphasize, multidisciplinary rounds and clinics are key. At our center, every breast cancer diagnosis is discussed in rounds with all treating subspecialties present. A generalized plan, including timing, is established, and the patient is seen the following week in a multidisciplinary clinic where breast surgeons, oncologists, and plastic surgeons coordinate their care. From diagnosis onward, each specialist remains actively engaged in the patient's journey, ensuring that timing targets are met.

A clear example of the importance of education and communication within the multidisciplinary team is the patient recommended for neoadjuvant chemotherapy. In this scenario, the medical oncologist understands that surgery should take place approximately 4 weeks after chemotherapy completion and is already aware of the immediate reconstruction plan. This ensures that the oncologist refers the patient back for surgical planning at least 1 month before chemotherapy concludes, allowing adequate time for preparation and scheduling.

Finally, patient navigation and communication tools, such as a patient navigator or an online treatment tracker, can further streamline coordination and prevent delays.^3,4^

While our center has prioritized education, communication, and navigation, we have not yet taken the critical step of measuring our success as the authors of this study have done. For this and their ongoing contributions, I applaud them.
